# Introgression Leads to Genomic Divergence and Responsible for Important Traits in Upland Cotton

**DOI:** 10.3389/fpls.2020.00929

**Published:** 2020-07-06

**Authors:** Shoupu He, Pengpeng Wang, Yuan-Ming Zhang, Panhong Dai, Mian Faisal Nazir, Yinhua Jia, Zhen Peng, Zhaoe Pan, Junling Sun, Liru Wang, Gaofei Sun, Xiongming Du

**Affiliations:** ^1^State Key Laboratory of Cotton Biology, Institute of Cotton Research, Chinese Academy of Agricultural Sciences, Anyang, China; ^2^College of Plant Science and Technology, Huazhong Agricultural University, Wuhan, China; ^3^Department of Computer Science and Information Engineering, Data Mining Institute, Anyang Institute of Technology, Anyang, China

**Keywords:** Upland cotton, interspecific hybridization, introgression, genomic divergence, maturity, fiber quality

## Abstract

Understanding the genetic diversity and population structure of germplasms is essential when selecting parents for crop breeding. The genomic changes that occurred during the domestication and improvement of Upland cotton (*Gossypium hirsutum*) remains poorly understood. Besides, the available genetic resources from cotton cultivars are limited. By applying restriction site-associated DNA marker sequencing (RAD-seq) technology to 582 tetraploid cotton accessions, we confirmed distinct genomic regions on chromosomes A06 and A08 in Upland cotton cultivar subgroups. Based on the pedigree, reported QTLs, introgression analyses, and genome-wide association study (GWAS), we suggest that these divergent regions might have resulted from the introgression of exotic lineages of *G. hirsutum* landraces and their wild relatives. These regions were the typical genomic signatures that might be responsible for maturity and fiber quality on chromosome A06 and chromosome A08, respectively. Moreover, these genomic regions are located in the putative pericentromeric regions, implying that their application will be challenging. In the study, based on high-density SNP markers, we reported two genomic signatures on chromosomes A06 and A08, which might originate from the introgression events in the Upland cotton population. Our study provides new insights for understanding the impact of historic introgressions on population divergence and important agronomic traits of modern Upland cotton cultivars.

## Introduction

The *Gossypium* genus (cotton) includes more than 50 species with wide distribution around the world (Wendel and Grover, 2015). Only four species from this genus have been domesticated during the history of cotton cultivation, including two diploid species (*G. herbaceum*, A_1_ and *G. arboretum* A_2_) and two tetraploid species (*G. hirsutum* (AD)_1_ and *G. barbadense* (AD)_2_), with *G. hirsutum* (Upland cotton) accounting for more than 95% of cotton fiber production in the modern world (Tyagi et al., 2014). The tetraploid cotton originated from a natural hybridization event resulting in merging of the A and D genomes, approximately 1–2 million years ago (Wendel et al., 2010). In addition to the two domesticated tetraploid species (*G. hirsutum* and *G. barbadense*), five other wild species are distributed in the Hawaiian Islands (*G. tomentosum*, (AD)_3_), Brazil (*G. mustelinum*, (AD)_4_), Galapagos Islands (*G. darwinii*, (AD)_5_), Dominican Republic (*G. ekmanianum*, (AD)_6_) and Wake Atoll (*G. stephensii*, (AD)_7_) (Percival et al., 1999; Grover et al., 2015; Gallagher et al., 2017). The suggested diversity-center of *G. hirsutum* is the Caribbean and Central America (southern Mexico and Guatemala), where seven geographical landraces have formed: *yucatanense*, *palmeri*, *morril*, *richmondi*, the extensively distributed races *punctatum*, *latifolium*, and *marie-galante*. The *yucatanense* race is considered as the most primitive form of *G. hirsutum*, and a subpopulation of *punctatum* was derived from this race (Wendel et al., 2010). *Palmeri*, *morrill*, and *richmondi* are three comparatively improved races distributed in several relatively small regions (Wendel et al., 1992; Percival et al., 1999). Modern elite cultivated *G. hirsutum* (Upland cotton) is reported to be derived from annual *latifolium* with better fiber quality due to lineage introgression from *G. barbadense* (Wendel et al., 2010).

DNA markers have been successfully applied in previous studies to exploit the cotton diversity and QTL mapping, majority of the molecular markers used in cotton depicted the low genetic diversity in modern cultivated Upland cotton germplasm (Brubaker and Wendel, 1994; Iqbal et al., 2001; Abdalla et al., 2001; Hinze et al., 2012; Fang et al., 2013; Tyagi et al., 2014; Fang et al., 2017; Wang et al., 2017; Ma et al., 2018). This genetic bottleneck impedes further gain in cotton improvement through conventional breeding techniques, especially considering future requirements for increased fiber quality and stress tolerance. Therefore, investigation and understanding of the genetic structure and elucidation of the genetic background of the existing Upland cotton germplasm are lager concern. Simple sequence repeats (SSRs) marker-based studies have been performed to investigate the genetic diversity of the Chinese Upland cotton germplasm (Chen and Du, 2006; Pang et al., 2006). However, these studies were limited by the sample size of the investigated population and molecular marker resolution, which resulted in an unclear depiction of the genetic background of Chinese Upland cotton germplasm. Advancement in the field of genomics, with the whole-genome sequence for cotton (Li et al., 2015; Zhang et al., 2015), lead to the rapid development of single nucleotide polymorphism (SNP) markers. Recently, most of the SNP-based studies focused on SNP marker development (Hulse-Kemp et al., 2015) and QTL detection (genetic map construction) primarily utilizing segregating populations (Wang S. et al., 2015; Zhang et al., 2016; Islam et al., 2016). Besides, genome-wide association studies were biased towards the identification of putative candidate genes for a subjective trait (Fang et al., 2017; Wang et al., 2017; Ma et al., 2018), whereas these studies lacked the incentive to provide basic information about genetic diversity of *G. hirsutum* population.

China is the world's largest cotton fiber producer and consumer (FAO, 2018). The early Chinese Upland cotton germplasm (introduced to Yangtze River and Yellow River regions) was mainly introduced from the United States and the former Soviet Union (Xinjiang province region). Cotton breeding programs in China resulted in the development of series of backbone parents with the integration of pedigree and hybrid breeding methods complemented by adaptation to the local environment (Liang et al., 2002). In addition, Chinese breeders utilized the wild relatives and landraces of *G. hirusutum* and generated abundant introgression lines to transfer favorable traits to commercial cultivars via interspecific hybridization (Liang et al., 2002). This endeavor extensively broadened the genetic pool of Chinese cotton germplasm and led to the further development of a series of elite lines with superior fiber quality and better stress response. However, the genetic basis of interspecific hybridization and its impact on the genomic structure and agronomic traits are still unknown.

A recent study, based on SNP microarray, demonstrated the presence of extensive genomic divergence in the Upland cotton source germplasm and suggested that divergent genomic regions might be related to maturity and heterosis (He et al., 2019). However, the origin of these regions is still unknown. Hereby, using RAD-seq technology, the genetic diversity of 582 tetraploid cotton accessions and their population structure was revealed. The origin of divergent genomic regions within cultivars was also confirmed. Through integration of the genome-wide association studies (GWAS) and previously reported QTLs, the agronomic contribution of variations within these regions was further discussed.

## Materials and Methods

### Plant Materials, Sampling and DNA Extraction

A total of 582 tetraploid cotton accessions, including 470 accessions representing most of the genetic diversity of the Chinese *G. hirsutum* cultivars, 105 accessions belonging to eight geographic landraces, four *G. barbadense* accessions and three accessions from wild relatives, were examined in this study. detailed information for the accessions is provided in **Supplementary Table S1**. All cultivars were planted in the experimental field of the Institute of Cotton Research, Chinese Academy of Agricultural Sciences, Anyang, China. The landraces and wild relatives were sampled from the National Wild Cotton Nursery, Sanya, China. DNA from all samples was extracted from young fresh leaves following the CTAB method described by Paterson (Paterson et al., 1993).

### Library Construction and Sequencing

Genomic DNA was first quantified on a Qubit 2.0 fluorometer (Invitrogen), after which the concentration was calculated. the DNA was diluted to 50 ng/μl; and 1 µg of each sample was transferred to a clean 200 μl PCR plate (Axygen). The genomic DNA in each well was digested with 1 μl of FastDigest TaqI (Fermentas) for 10 min at 65 °C in a volume of 30 μl. For the ligation reaction, 1 μl of barcoded adapters (10 μM) was added to individual wells, together with T4 DNA ligase (Enzymatics), in a total volume of 40 μl. The ligation reaction was incubated for 1 h at 22 °C and then heat-inactivated at 65 °C for 20 min. Twenty-four ligation products from different samples were pooled into a single tube, and 2 µl of chloroform was added to inactivate the restriction enzyme. The mixtures were subsequently centrifuged at 12,000 rpm for 1 min, and the supernatant was transferred to a new tube. DNA fragments between 400 and 700 bp in length were screened in 2% agarose gels (Amresco) and purified using a QIA quick Gel Extraction Kit (QIAGEN). Next, the samples were resuspended in 50 µl of elution buffer and amplified via 10 cycles of PCR. Each amplification reaction included 8 µl of the library, 25 µl of Phusion Master Mix (Phusion high-fidelity, Finnzymes), 1 µl of the common primer (10 µM), 1 µl of the index primer (10 µM) and 15 µl of water. The amplified library was purified using a QIA quick PCR Purification Kit (QIAGEN), then quantified on an Agilent2100 Bioanalyzer (Agilent) and sequenced on an Illumina Hiseq2000 instrument (Illumina), as per the manufacturer's protocol.

### Variant Calling

In this study, approximately 0.74 billion clean reads of 85 bp in length (597 GB) were generated from the Illumina platform. We used BWA (ver. 0.7.12) (Li and Durbin, 2009) to map all clean reads on the *G. hirsutum* genome (Zhang et al., 2015) with default parameters; only paired-end reads that were both mapped to the genome were retained for variant calling. SAMtools (ver. 1.1) (Li et al., 2009) was used for variant calling. First, reads with a quality of less than 20 were discarded, after which reads that passed quality filtering were assigned to call variants using ‘bcftools’; (ver. 1.1); the ultimate SNP set (total 253,679 SNPs) was further filtered using the ‘vcfutils.pl’ script of ‘bcftools’;, with the following parameters: -Q 10 -d 2 -D 2000 -a 2 -w 3 -W 10 -1 0.0001 -2 1e-100 -3 0 -4 0.0001 -e 0.0001.

### Phylogenetic Tree Construction and Population Genetic Analysis

A total of 9,868 SNPs screened from the set of 253,679 SNPs (MAF >0.05, Missing <0.1, and heterogeneity <0.3) were employed to construct the phylogenetic tree for all accessions using FastTree software (Price et al., 2009). We further screened out another SNP set of 68,118 SNPs from the 253,679 SNP set (MAF >0.05, Missing <0.2 and heterogeneity <0.3) to analyze population structure using ADMIXTURE software (Alexander et al., 2009). Principle component analysis (PCA) was performed using the ‘smartpca’ module of EIGENSOFT (https://www.hsph.harvard.edu/alkes-price/software/). The nucleotide diversity (π) was calculated by VCFTools (Danecek et al., 2011).

### Physical Localization of SSR Markers and QTLs in the Genome

A total of 65,412 raw SSR marker clones were downloaded from COTTONGEN (www.cottongen.org). The sequences were aligned against the *G. hirsutum* genome (Zhang et al., 2015) using Blastn (ver. 2.2.30) (McGinnis and Madden, 2004). Only the two results (A and D subgenomes) with the smallest *p* values were retained as the possible physical positions of the SSR markers. For physical localization of the previously reported QTLs (references were listed in **Supplementary Tables S4** and **S7**), only the positions (genetic map chromosome assignment (Wang et al., 2006) of QTLs that agreed with the physical position of their corresponding SSR markers (genome chromosome assignment) were retained for further analysis. When two flanking markers were not located on the same chromosome, only markers that agreed with the genetic map were retained to represent that QTL.

### Phenotyping and GWAS

All phenotypic data including development stage (maturity), boll weight, lint percentage, seed index, fiber length, fiber strength, micronaire and fiber elongation rate were investigated followed by “Descriptors and data standard for cotton” in three typical cotton-growing regions of China, including Anyang (Yellow River region), Nanjing (Yangtze River region) and Akesu (Xinjiang) for three years (2007–2009) with three replications for each environment. GWAS was performed by standard EMMAX procedure described by Kang et al. (2010) (http://genetics.cs.ucla.edu/emmax/).

### Introgression Analysis

To identify introgression in Upland cotton cultivars, (1) the donor group was screened according to the result of population structure analysis (when *K* = 4) (selected from Group-0). Accessions in Group-0 containing a “deep blue” or “purple” lineage were selected as donor-group-blue (total of 50 accessions) or donor-group-purple (total of 73 accessions). (2) The receptor accessions were any cultivar that may have contained an introgressive fragment (selected from Group-1, Group-2 and Group-3). At each site for any given receptor accession, we first calculated the site identical sample count (site ISC) by comparing its genotype with the donor group (deep blue and purple were calculated separately). For instance, at one site, if the genotype of the receptor accession was “A” and donor group contained 20 “As”, then the site ISC was recorded as 20 for that receptor accession. The window ISC was the sum of all site ISCs in the 1 Mb window region. Second, the window valid sample count (window VSC) was recorded as the total number of samples with known genotypes. Finally, the introgressive index was calculated using the following formula:

$$\text{Introgressive}\;\text{index}=\frac{\text{windowed}\;\text{ISC}\;\text{of}\;\text{donor}\;\text{group}}{\text{windowed}\;\text{VSC}\;\text{of}\;\text{donor}\;\text{group}}-\frac{\text{windowed}\;\text{ISC}\;\text{of}\;\text{acceptor}\;\text{group}}{\text{windowed}\;\text{VSC}\;\text{of}\;\text{acceptor}\;\text{group}}$$

All introgressive indexes larger than 0.15 were screened as introgression fragments and plotted in **Figure 3**.

## RNA-Seq Data Manipulation

All RNA-seq data were downloaded from NCBI and were first mapped on the genome using TopHat (ver. 2.0.13) (Trapnell et al., 2009), after which the expression level was calculated (fragments per kilobase of transcript per million mapped fragments, FPKM) using Cufflinks (Trapnell et al., 2010). The transcriptome data used in this study came from various tissues of TM-1 (PRJNA248163) and 10 DPA and 20 DPA fibers of *yucatanense* (SRP017061).

## Results

### Genetic Relationships, Population Structure, and Genomic Divergence of Tetraploid Cotton

**Figure 1:**
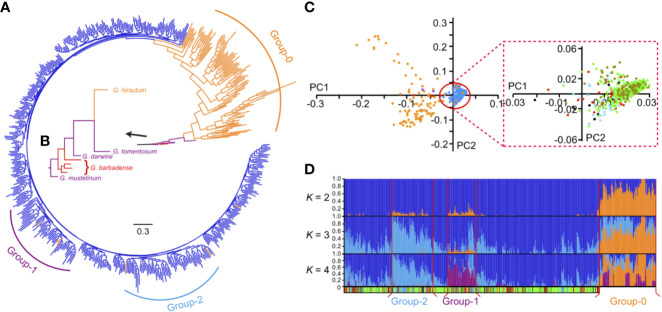
The phylogenetic tree and genetic structure of tetraploid cotton. **(A)** The phylogenetic tree of all tetraploid cotton accessions. Three distinct groups are marked as purple (Group-1), blue (Group-2) and orange (Group-0) bars. **(B)** The zoomed view of wild tetraploid cottons and *G. barbadense* branches. Wilds (purple), *G. barbadense* (red) and *G. hirsutum* (orange) branches are marked with different colors, respectively. **(C)** The principal components analysis (PCA) of all accessions. First two principal components were used as *x* (PC1) and *y* (PC2) axis to plot 582 accessions in this study. In left panel, dots with different colors indicated wild species (purple), *G. barbadense* (red), *G. hirsutum* landraces (orange) and *G. hirsutum* cultivars (blue), respectively. In right panel, the red box indicated the zoomed view of PCA plots within *G. hirsutum* cultivars, different ecotypes including central Asia (deep blue), US (red), Yellow River region of China (green), Yangtze River region of China (asparagus) and other nations (black) were represented by dots with different colors. **(D)** The model-based clustering analysis with different clusters (*K* = 2 to 4). The y axis quantifies cluster membership, and the x axis represents the accessions; the order is identical with phylogenetic tree (the root was on the right). Group-1, Group-2, Group-0 and Group-3 (all remaining cultivars) are separated by vertical red lines. The colored bar at the bottom indicates the geographical origin of all accessions, light blue, Central Asia; red, United States; green, Yellow River region of China; asparagus, Yangtze River region of China; black, other nations; white, unknown.

The whole sequencing panel contained 470 cultivars, 105 landraces, four *G. barbadense* accessions, and three wild relatives. Most of the cultivars were collected from China, and nearly all the landraces were from the United States, except *G. purpurascens* (**Supplementary Table S1**). After SNP filtering (MAF >0.05, missing <0.1, heterogeneity <0.3), we used 9,868 SNPs extracted from a raw SNP set (253,670 SNPs) to construct the phylogenetic tree. *G. mustelinum*, a wild and primitive tetraploid cotton species distributed in a small region of northeastern Brazil (Brubaker and Wendel, 1994), was used as the root for the phylogenetic tree (**Figure 1A**). Four *G. barbadense* cultivars were clustered together next to *G. mustelinum*, followed by two island species viz. *G. darwinii* and *G. tomentosum*. *G. hirsutum* clade was positioned next to *G. tomentosum* (**Figure 1B**). Almost all of the landraces (**Figure 1A**, orange branches) were separated from the clades of wild species earlier than the cultivars (**Figure 1A**, blue branches). We defined landraces together with the wild species and *G. barbadense* as “Group-0” (**Figures 1A, B**, red, purple, and orange branches). Remaining accessions belonged to *G. hirsutum* lines or cultivars (**Figure 1A**, blue branches), most of them (383 of 470, **Supplementary Table S1**) were elite genetic materials or commercial cultivars collected from various locations in China. In the phylogenetic tree, landraces could easily be distinguished from cultivars. However, random distribution of cultivars in this clade was in contrast with the geographic distribution of them (**Supplementary Table S1**) Overall, the phylogenetic tree showed that the cultivars branch was relatively compact than the landraces branch. Principal component analysis (PCA) complemented the compactness of the cultivars branch (**Figure 1C**). The whole-genome average nucleotide diversity (π) of landrace group (0.041 × 10^−3^) was also greater than cultivars (0.022 × 10^−3^). Therefore, we concluded that the genetic diversity of *G. hirsutum* landraces was higher than cultivars. The narrow genetic diversity of cultivars might have been caused by the limited number of early parentages.

Interestingly, in the model-based clustering analysis, Group-0 appeared to harbor two kinds of mixed lineages (**Figure 1D**, orange and blue, when *K* = 2), but the cultivar group was biased toward one of them (**Figure 1D**, blue, when *K* = 2). Among the cultivars, in particular, two distinct groups (**Figure 1D**, named as Group-1 and Group-2 harbored relatively more exotic introgression components (orange, when *K* = 2) than other accessions. Group-1 and Group-2 contained 54 accessions (including three landraces) and 76 accessions (including two landraces), respectively. The remaining 346 accessions (including one landrace) comprised Group-3 (**Supplementary Table S1**). Furthermore, Group-2 and Group-1 appeared to exhibit different lineage compositions at *K* = 4: more purple lineages were present in Group-1, whereas more blue lineages were observed in Group-2 (**Figure 1D**). According to the germplasm source information (**Supplementary Table S1**), we found that the purple lineage (Group-1) contained the accessions mostly collected from Yellow and Yangtze River regions. The blue lineage (Group-2) were mainly collected from high-latitude regions, including 33 accessions from the Chinese Yellow River region and 22 accessions from the former Soviet Union, Xinjiang Province and Liaoning Province of China. These accessions showed several typical characteristics, such as early maturity with small and compact plant architecture.

### The Phenotypic Characteristic of Two Sub-Groups of *G. hirsutum* Cultivars

**Figure 2:**
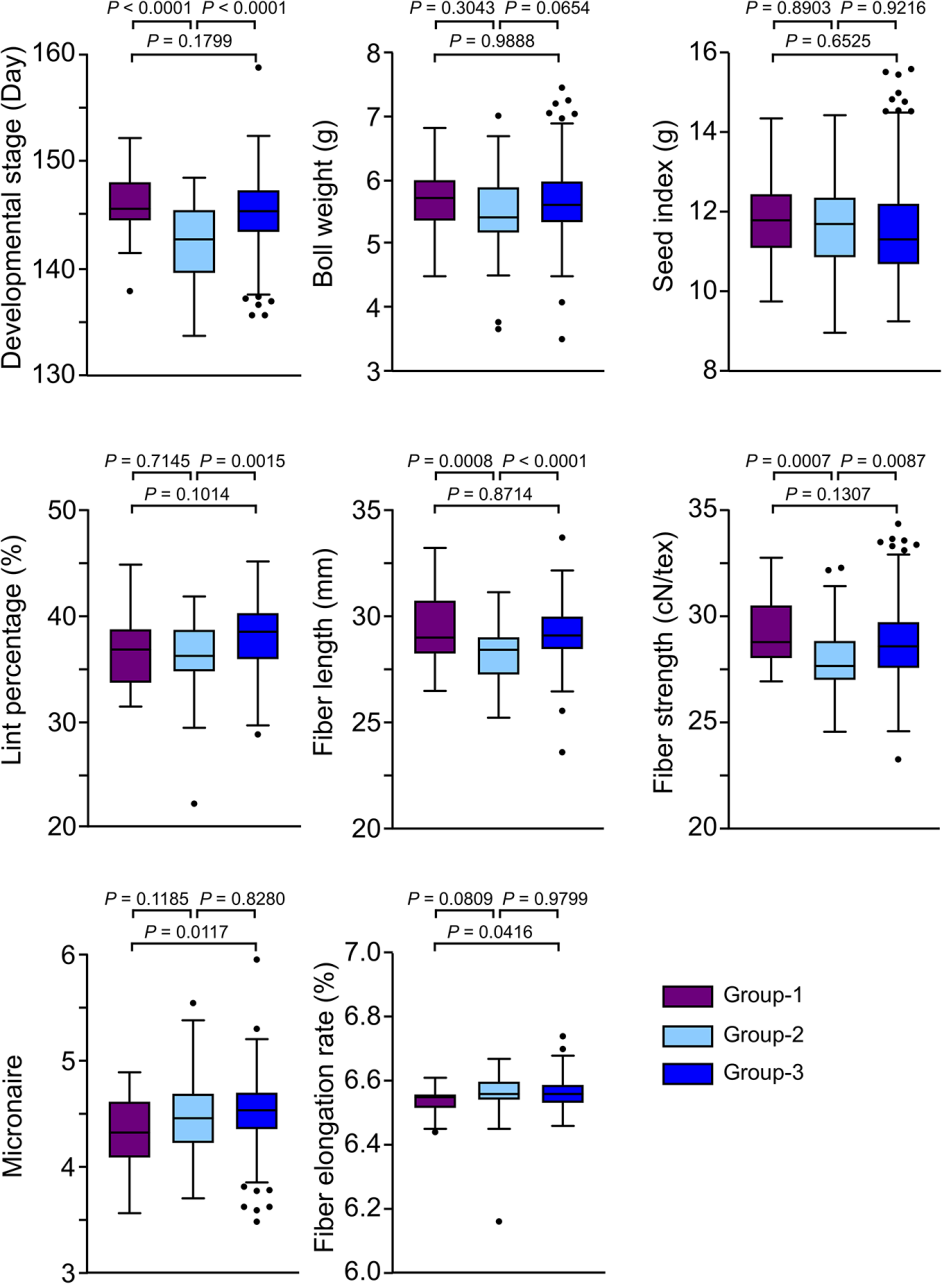
Comparison of investigated traits among groups. In the box plots, the centerline, box limits, and whiskers indicate median, upper and lower quartiles, and 1.5× interquartile range, respectively. Points show outliers. The significances were tested by Tukey's multiple comparisons test.

To investigate the phenotypical variation among three sub-groups, we analyzed eight major agronomic traits including maturity (development stage), yield (boll weight, seed index, and lint percentage), fiber quality (fiber length, fiber strength, micronaire, and fiber elongation rate) in three locations (Anyang, Nanjing, and Akesu) for three cropping seasons. Descriptive statistics for maturity significantly differentiated three groups, while Group-2 demonstrated early maturity than other groups. (**Figure 2**). Moreover, both Group-1 and Group-3 showed significantly better fiber quality (fiber length and fiber strength) than Group-2 (**Figure 2**). We also found that the fiber strength and micronaire of Group-1 were slightly better than Group-3. These results suggested that the accessions in Group-1 and Group-2 exhibited excellent fiber quality and significant early maturity, respectively.

### Genomic Divergence and Introgression in the Upland Cotton Population

**Figure 3:**
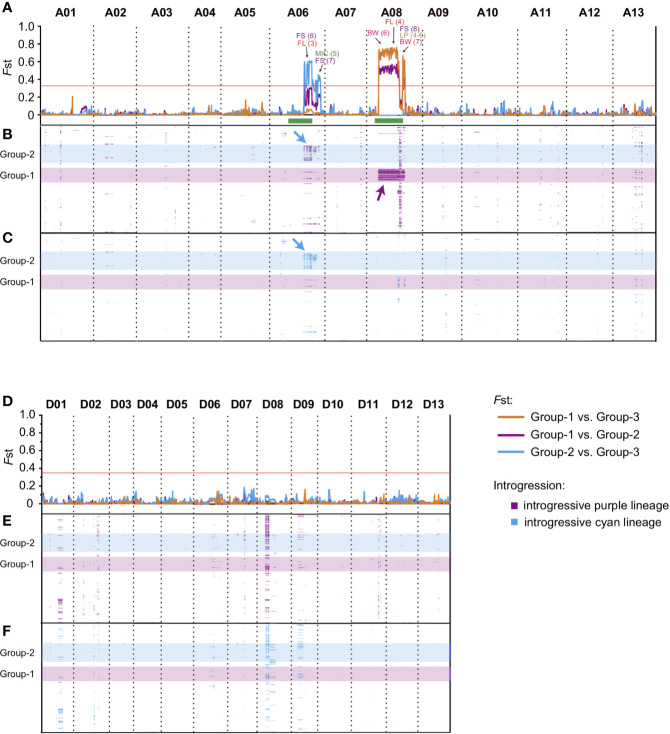
The differentiation and introgression within cultivar population. The divergent genomic regions on At subgenome **(A)** and Dt subgenome **(D)** within Upland cotton cultivars. The *y* axis indicates the *F*st value, and three comparisons of Group-1 vs. Group-3 (orange), Group-2 vs. Group-3 (light blue) and Group-1 vs. Group-2 (purple) are represented by lines with different colors, respectively, the horizontal red lines represent the threshold value of *F*st (top 1%, *F*st >0.364), and the regions above lines indicates the divergent genomic regions within Upland cotton cultivars. QTLs corresponding to different traits were marked by different colors; the number in parentheses indicates the QTL IDs (detailed information of QTLs are listed in **Supplementary Table S3**). BW, boll weight; SI, seed index; LP, lint percentage; FL, fiber length; FS, fiber strength; MIC, micronaire. The green bars at the bottom of Chr. A06 and A08 indicate their putative pericentromeric regions (Wang S. et al., 2015). The introgression regions (introgression index >0.15) derived from “purple” **(B, E)** and “light blue” **(C, F)** lineages were presented by purple and light blue band, y-axis of **(B)**, **(C)**, **(E)**, and **(F)** indicated the cultivars, the order of accessions were consistent with cluster result (**Figures 1A, B**). The position of Group-1 (light blue) and Group-2 (purple) were highlighted by a transparent band. The major introgression fragments on A06 (blue) and A08 (purple) were marked by arrows. All QTL references were listed in **Supplementary Table S3**.

In the present study, two distinct sub-groups (Group-1 and Group-2) that contained relatively more exotic introgressions were identified through ancestry analysis (**Figure 1D**). To further investigate the specific introgressed genomic regions, we calculated the pairwise population differentiation statistic (*F*st) for Group-1 vs. Group-3, Group-2 vs. Group-3, and Group-1 vs. Group-2 (Group-3 comprised remaining cultivars or lines except Group-1 and Group-2). The highly divergent regions (top 1%, *F*st >0.364) identified among these groups were located in two regions, ranging from approximately 63.9 to 94.9 Mb on chromosome A06 and 21.8 to 71.7 Mb on chromosome A08 (**Figures 3A, D**, **Supplementary Table S2**). The genomic differentiation of these regions was dramatically higher than the average whole-genome level, indicating that they were the major genomic divergence regions within the *G. hirsutum* cultivar population. We identified 9, 3 and 3 highly differentiated regions on A06 in the comparison of Group-2 vs. Group-3 (average *F*st = 0.437), on A08 for Group-1 vs. Group-3 (average *F*st = 0.617) and for Group-1 vs. Group-2 (average *F*st = 0.467), respectively (**Figures 3A, D**, **Supplementary Table S2**). A total of seven QTLs related to both yield and fiber quality were located in the divergent region (**Supplementary Table S3**). Interestingly, we found that most of these QTLs were derived from a parent with explicit wild introgression, such as *G. anomalum*, *G. barbadense*, or *G. arboretum* (**Supplementary Table S3**), which strongly suggested that these genomic regions could be related with genetic introgression during interspecific hybridization.

To confirm the genomic distribution of introgressive fragments in the cultivar population, by using Group-0 as a donor population (**Figure 1C**, when *K* = 4), we further calculated the distribution of the introgression index for the “purple” and “light blue” lineages of each accession on the At sub-genome (**Figures 3B, E**) and Dt sub-genome (**Figures 3C, F**), respectively. For all the cultivars, the introgressive fragments derived from Group-0 were mainly located on chromosomes A06, A08, D01, D08, and D09 (**Figures 3B, C, E, F**). In the two distinct groups, Group-1 carried “purple” lineage on A08 (**Figure 3B**, indicated by purple arrows), and Group-2 carried both “purple” and “light blue” lineages on A06 (**Figures 3B, C**, indicated by blue arrows). These unevenly distributed distinct introgressive fragments in Group-1 and Group-2 might be the cause of population differentiation in Upland cotton cultivars. We further found that the genotypes of Group-1 and Group-2 presented high heterozygosity and missing alleles in their specific introgression regions (A06 and A08) (**Supplementary Figure S4**). Besides, we found that these regions were mainly located at predicted pericentromeric regions on A06 and A08 (green bars at the bottom of **Figure 3A**) (Wang S. et al., 2015). In these regions, some QTLs related with yield and fiber quality were also detected in previous studies (**Figure 3A**, **Supplementary Table S3**). Therefore, these megabase-size regions with strong genetic linkage disequilibrium might have resulted from the low recombination frequency of pericentromeric regions.

### The Potential Function of Divergence Regions on Chromosomes A06 and A08

Considering the specific pedigrees and phenotypes of the accessions in Group-1 and Group-2 with distinct genotypes showed exceptionally high divergence and harbored various QTLs (**Figures 2** and **3**), we speculated that the genomic variations in regions might be associated with important traits. According to the gene annotations, a total of 282 and 289 genes were annotated in the two regions on A06 and A08, respectively (**Supplementary Tables S4** and **S5**). Some genes in these regions showed different expression patterns in various tissues. We further screened some genes with tissue-specific expression that might regulate ovule or fiber development (being highly or expressed explicitly in ovules or fiber (**Supplementary Figures S5** and **S6**). For instance, several functionally characterized genes related to fiber, trichome, or root development were explicitly expressed in ovules or fibers. On chromosome 6, *meristem layer 1* (*ML1*, A06G1283) encoding a homeobox protein similar to GL2, is a transcription factor that interacts with MYB25 to further regulate trichome development in *Arabidopsis* (Zhang et al., 2010) and cotton (Ding M. et al., 2015). *β-ketoacyl-[acyl carrier protein] synthase I* (*KAS1*, A06G1195) is a crucial gene to regulate root development in rice (Ding, W. et al., 2015) and *1-Aminocyclopropane-1-Carboxylic acid Oxidase 4* (*ACO4*, A06G1341) is responsible for ethylene production and therefore influences cotton fiber growth (Qin et al., 2007) (**Supplementary Table S4**, **Supplementary Figure S5**). On chromosome A08, *glycosyl hydrolase 9C2* (*GH9C2*, A08G0869) is a gene that impacts cell wall development in plants (Glass et al., 2015). *MYB103* (A08G0993) is specifically expressed in 20 DPA and 25 DPA fibers and has been suggested to affect secondary cell wall biosynthesis and deposition (Sun et al., 2015) (**Supplementary Table S5**, **Supplementary Figure S6**). We also identified several putative genes that might be related to fiber development, i.e. the *cytochrome P450*, *family 77*, *subfamily B*, *polypeptide 1* (*CYP77B1*, A06G1290) and *RPM1 interacting protein 4* (*RIN4*, Gh_A06G1343) genes and an unannotated gene (GhA08G1000); these genes also showed specific expression patterns in ovules and fiber tissues (**Supplementary Table S5**, **Supplementary Figures S5** and **S6**). The causal nucleotide variations in these regions (or on genes) and their genetic functions in cotton should be further verified.

### Genome-Wide Association Study (GWAS) Further Confirmed the Function of Variations in Divergence Regions on Two Chromosomes

**Figure 4:**
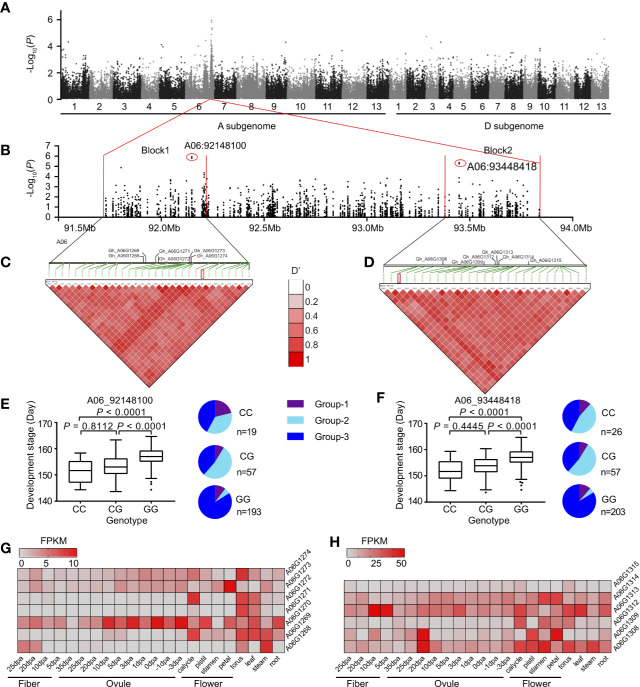
The maturity trait-associated loci in the divergence region on chromosome A06. **(A)** Manhattan plots of GWAS for the development stage (Nanjing-2009). **(B)** the local Manhattan plot for the signals on chromosome A06, two strongest signals (A06_92148100 and A06_93448418) were marked by red circles. **(C, D)** The LD heatmap and annotated genes in two blocks. The location of the strongest signals was labeled by red rectangles. **(E, F)** Box plots for the development stage, according to the genotype of two strongest signals A06_92148100 (left) and A06_93448418 (right). In the box plots, the centerline, box limits, and whiskers indicates median, upper and lower quartiles, and 1.5× interquartile range, respectively. Points show outliers. Significances were tested by Dunn's multiple comparison test. The pie charts indicated the sub-group categorization of the strongest signals in GWAS population. The heatmaps indicated the level of genes in block 1 **(G)** and block 2 **(H)**, respectively. FPKM, fragments per kilobase per million.

To further confirm the genetic function of divergence regions on chromosomes A06 and A08, we performed GWAS on 316 representative accessions selected from the whole panel of the population. We mainly focused on the significant GWAS signals (−logP >4) in the divergence regions on chromosomes A06 and A08. Interestingly, for chromosome A06, the majority of signals were associated with lint percentage (33/83) and development period (maturity) (17/83). However, for chromosome A08, the signals were associated with fiber quality traits, such as fiber strength (44/101) and fiber length (19/101) (**Supplementary Table S6**). Moreover, two adjacent LD blocks were identified in the overlapped regions between GWAS (development stage) and population divergence (*F*st) (**Figures 4A, B**). In these regions, a total of 12 genes were detected in block 1 (491 Kb) and block 2 (401 Kb) (**Figures 4C, D**), respectively. Furthermore, the allelic frequencies of signals A06_92148100 and A06_93448418 showed that Group-3 carried much more GG allele than Group-2 (**Figures 4E, F**). The development stage of accessions carrying genotype CC (92,148,100 and 93,448,418) showed significantly early maturity than other two genotypes (**Figures 4E, F**). In these regions, several genes such as *A06G1269*, *A06G1272*, *A06G1309*, and *A06G1314* were specifically expressed in floral organs (**Figures 4G, H**). These genes might control floral development and further regulating cotton maturity. In A08, a total of three similar blocks were detected associated with fiber strength (**Supplementary Figure S3**). We detected several genes in these blocks, possibly associated with fiber strength. For instance, *A08G0929* in block 2, located nearby the strongest signal of this block (A08_59869122), showed gradual increase in expression level from 10 DPA to 25 DPA in fiber. This gene involved in the flavonol and lignin biosynthetic process in *Arabidopsis* (Moinuddin et al., 2010) and *Brachypodium* (Ho-Yue-Kuang et al., 2016), which are two major biological processes to determine fiber quality in cotton. Taking into account the ecological distribution and morphological characteristics of three subgroups, our results emphasized the correlation of highly differentiated regions on chromosomes A06 and A08 with maturity and fiber strength, respectively.

## Discussion

### Complex Genetic Background of the *G. hirsutum* Landrace Population and Narrow Diversity of the Cultivars Population

In this study, the relationship observed among wild tetraploid cottons, *G. barbadense*, and *G. hirsutum* cotton was consistent with a previous study based on SSR markers (Lacape et al., 2006). According to the phylogenetic tree and genetic structure analysis, within Group-0 (**Figures 1A, D**), the principal branch of the *marie-galante* race was the first to separate from wild species (except for one *morrill* accession). This landrace has widespread along the coast from southern North America to central South America and suggested to have been derived from introgression between *G. hirsutum* and *G. barbadense* (Wendel et al., 2010). Our results confirmed that *marie-galante* is the *G. hirsutum* landrace closest to *G. barbadense* and represents a potential source for increasing the diversity of improved lines in future cotton breeding. The *punctatum* and *latifolium* races include both perennial and annual forms and are widely distributed across Central America (Hutchinson et al., 1947). This extensive geographical distribution and frequent human interventions provided more opportunities for intercrossing with other indigenous races to increase the genetic variation of these races. Therefore, in the phylogenetic tree generated in the present study, these races were distributed in Group-0 (**Figures 1A, B**). Furthermore, these two races have been suggested to be the most probable original races of modern Upland cotton (Wendel et al., 1992). We also found that five *latifolium* accessions clustered in the cultivar clade (**Figure 1A**) (a total of six accessions clustered in the cultivar clade, including one *marie-galante* accession), implying that genetic background of *latifolium* accessions is very close to modern cultivars and most of Chinese Upland cotton germplasm was possibly originated from *latifolium*.

In the present study, both the phylogenetic tree and genetic structure analyses demonstrated the complex genetic background of *G. hirsutum* landraces. Although some of these landraces can be phenotypically differentiated, there are no apparent genetic characteristics for clearly distinguishing each race. This situation is very likely caused by the overlap in the habitats of these landraces and human activities, resulting in landraces that are genetically mixed (especially in three geographically widely distributed races: *marie-galante*, *punctatum*, and *latifolium*) containing true wild, feral and cultivated populations (Coppens d'Eeckenbrugge and Lacape, 2014). Based on SNP markers, we were able to classify the landraces into different subtypes for a better understanding of their genetic background and further utilization in breeding programs.

Initial reports concerning domestication of Upland cotton suggested that *G. hirsutum* cultivars should have an abundance of primitive gene-pool (Shands et al., 1991). However, narrow genetic diversity of cotton cultivars has been noted in several previous studies (Liang et al., 2002; Chen and Du, 2006; Lacape et al., 2006). This paradox might be the result of domestication bottleneck i.e., selection for early maturity which led to loss of several elite alleles and favorable genes during the expansion of Upland cotton into North America (Chen et al., 2006). Our results were consistent with lower diversity in the cultivar population than in landraces (**Figure 1A**), implying that there is great potential for improving modern cotton cultivars by utilizing landraces. Further classification and exploitation of these landraces, with the utilization of high throughput phenotyping and genotyping, can be useful to shed light on the domestication history of *G. hirsutum* leading towards improved future cotton breeding programs.

### Genomic Differentiation Resulting From Landrace Introgression on Chromosomes A06 and A08 Was Responsible for Important Traits in Upland Cotton Cultivars

Nearly all modern cotton cultivars were primarily developed in the United States from four basic types (Acala, Plains, Delta, and Eastern type), which were originated from a diverse gene-pool (Petit Gulf) mixed with *G. hirsutum* and *G. barbadense* lineages (Shands et al., 1991). These four types of cotton were subsequently introduced to other major cotton production areas worldwide. American Upland cotton accessions were introduced in central Asian countries during the 1870–1880s. The initial germplasm was selected from a mixture of the American early-maturity varieties, including King, Triumph, and Russell's (Abdullaev and Abdullaev, 2013). Some elite varieties with excellent comprehensive characteristics (such as ‘108F’; and ‘Tashkent series’) were developed before regular breeding programs were established in the early 20th century (Abdullaev and Abdullaev, 2013). Two major ecotypes of Upland cotton were introduced into China in the early stage. The backbone parents of the central Asia type, exhibiting early maturity characteristics, were initially introduced into the northwestern and northeastern regions of China from the former Soviet Union (‘King’; cultivar). For the other two traditional cotton production regions in China (the Yangtze River and Yellow River regions), early varieties were directly selected and developed from American commercial varieties such as Stoneville and Deltapine series exhibiting broad adaptation (Shands et al., 1991). Due to environmental differences among regions, local varieties subsequently developed the corresponding features for adaptation to the local environment. In brief, the introduction of central Asian-type cotton primarily contributed early maturity-related genetic resource, while the Stoneville/Deltapine germplasm contributed extensive adaptability to modern Chinese Upland cotton cultivars.

In this study, the genetic clustering showed no distinct geographic patterns (**Figure 1D**), which might be due to the extensive environmental adaptability and frequent germplasm exchanges among regions during the breeding process. Previously, we have identified several divergent genomic regions on chromosomes A06 and A08 related to maturity and heterosis, respectively (He et al., 2019). In this study, we further confirmed these divergent regions in a larger population. More importantly, through introgression analysis, these large-scale variations were also detected in the landraces of *G. hirsutum*. Therefore, we suggested these variations in cultivars might have resulted from introgression of landraces and wild relatives. According to their geographic information, we found two distinct groups (Group-1 and Group-2) containing different introgressed genetic components that might be responsible for their different environmental adaptation. Our GWAS results further confirmed the specific haplotypes (or genes) on A06, which might regulate maturity in the cotton population (**Figure 4**). Although the origin of the accessions in Group-1 was mixed, according to the pedigree, wild or landrace lineage introgressed fragments could be identified in most of these accessions. In contrast to Group-2, there were more superior fiber accessions from the Yangtze River region (YZR) in Group-1. Therefore, we concluded that these two regions might be the significant genomic signatures for distinguishing Upland cotton germplasm adapted from different regions in China.

Interspecific hybridization breeding has played a critical role in the cotton breeding history worldwide; via hybridization among various *Gossypium* species, abundant introgression lines carrying excellent traits (i.e., high disease resistance, superior fiber quality) have been developed over the past decades (Liang et al., 2002). According to the GWAS results, we found that some regions (or genes) on A08 were strongly associated with fiber strength (**Supplementary Figure S3**).

Based on the results of genetic structure analysis (**Figure 1C**), population differentiation and introgression analyses (**Figure 3**), we clarified that the different introgressive components located on A06 and A08 might not only represent the major forces driving population differentiation in Upland cotton cultivars but also impact the major agronomic traits. In the breeding practices, introgression lines carrying excellent traits are often associated with certain disadvantages, such as superior fiber quality being associated with late maturity and a reduced yield; this phenomenon results from a linkage block region harboring antagonistic or pleiotropic genes. In tomato, co-localization of QTLs controlling multiple traits resulted from pericentromeric introgression from wild species (Budiman et al., 2004; Haggard et al., 2015). The repression of recombination in the pericentromeric region makes it difficult to precisely map the significant QTLs within this region (Haggard et al., 2015). Based on our results, the proportion of genotypes responding for early maturity and excellent fiber strength was declined in Group-3 (which was represented for most of the modern Chinese Upland cotton population) (**Figure 4F**). Therefore, considering the extensive range of introgressive regions on the chromosome, we conjecture that some major exotic QTLs controlling contrasted traits (i.e., early maturity and poor fiber quality) might be located at the same pericentromeric regions on A06 or A08 with strong linkage disequilibrium, resulting in the decrease proportion during the breeding process. According to genome annotations, although these regions span more than 100 Mb (**Supplementary Table S2**) in total, fewer than 300 genes are found in these regions on each chromosome. We also identified some genes that were specifically expressed in the ovule or fiber, which strongly suggested that they regulate fiber development (**Supplementary Tables S5** and **S6**, **Supplementary Figures S5** and **S6**). In the future, a comprehensive approach could potentially result in breaking the linkage utilizing functional genomics complemented by hybridization and make these genes into the application as a future breeding tool. Therefore, based on GWAS results and previously identified QTLs overlapping these regions, we strongly suggest that these regions are typical genomic signatures representing introgression lines in Upland cotton cultivars and genes in these regions are essential resources worthy of future study.

## Data Availability Statement

The datasets presented in this study can be found in online repositories. The names of the repository/repositories and accession number(s) can be found below: https://www.ncbi.nlm.nih.gov/, PRJNA353524.

## Author Contributions

XD, Y-MZ, and SH conceived and designed the experiments. PW, HX, ZPe, and ZPa performed library construction and sequencing. YJ, JS, and LW collected the field data. GS and PD performed the bioinformatics analysis. SH and GS analyzed the data. SH wrote the paper. MN edited the paper. All authors contributed to the article and approved the submitted version.

## Funding

This work was supported by grants from the National Key Research and Development Program of China (2016YFD0100306, 2016YFD0100203) and the National Natural Science Foundation of China (31871677).

## Conflict of Interest

The authors declare that the research was conducted in the absence of any commercial or financial relationships that could be construed as a potential conflict of interest.
