# Supplementary material for: Introgression Leads to Genomic Divergence and Responsible for Important Traits in Upland Cotton
Source: Front Plant Sci. 2020 Jul 6;11:929. doi: 10.3389/fpls.2020.00929 (PMC7381389; doi:10.3389/fpls.2020.00929)
Supplement: Supplementary file 7 [file Image_1.pdf]

Supplementary Figures

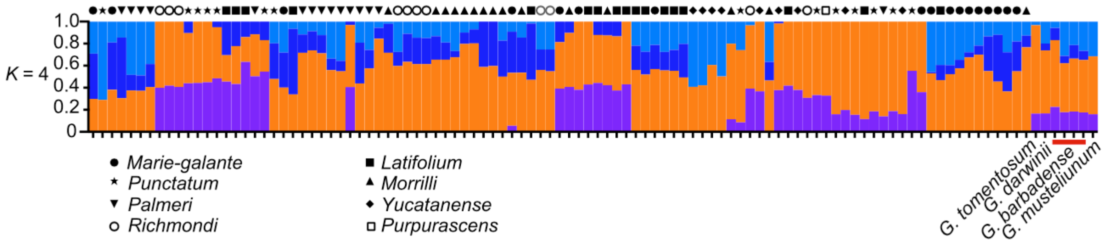

Supplementary Figure 1 Zoomed view of Group-0 based on structure analysis (when $K = 4$).

Different landraces are represented by different symbols.

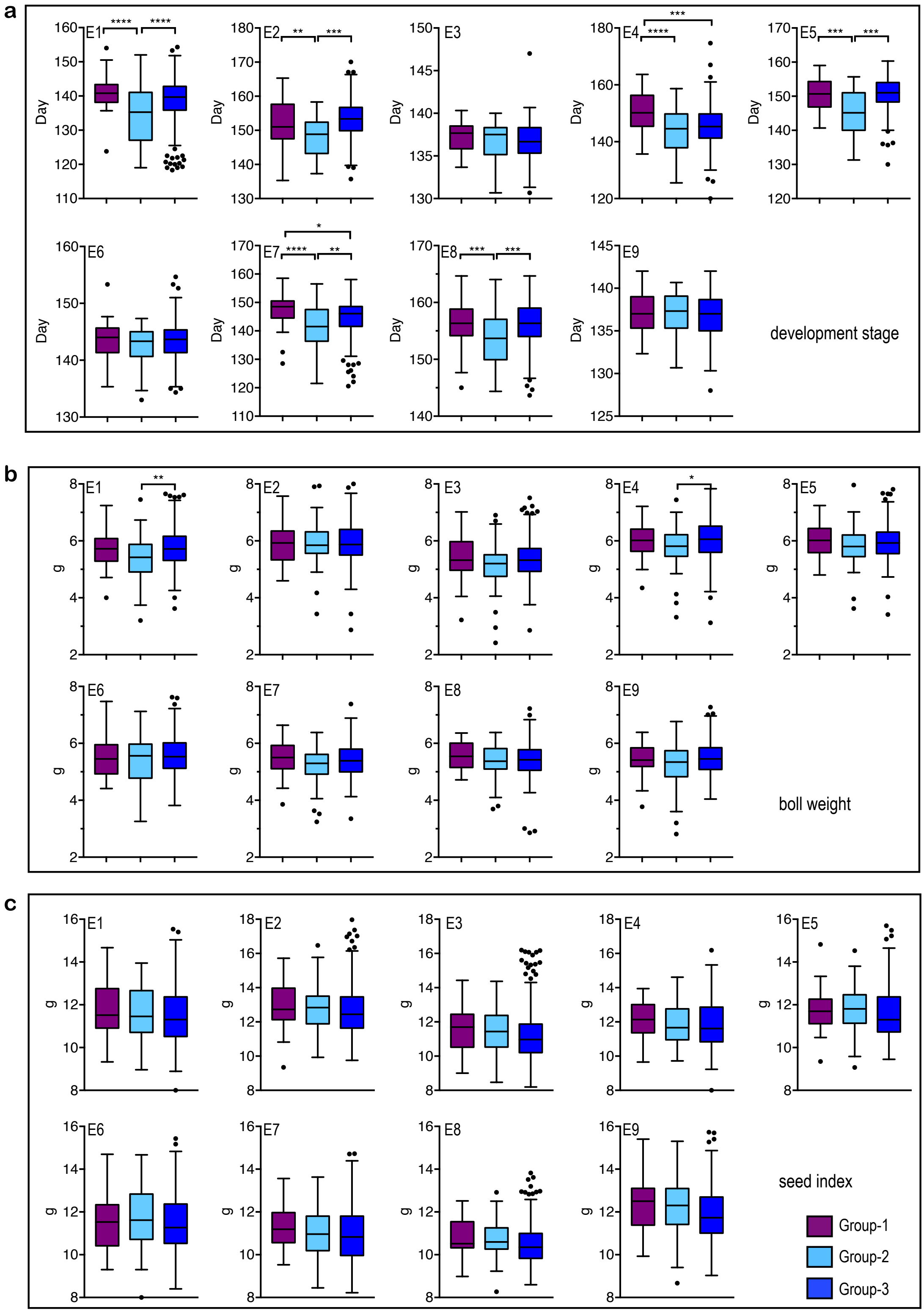
a
b
c
E1
E2
E3
E4
E5
E6
E7
E8
E9
Day
g
development stage
boll weight
seed index
Group-1
Group-2
Group-3

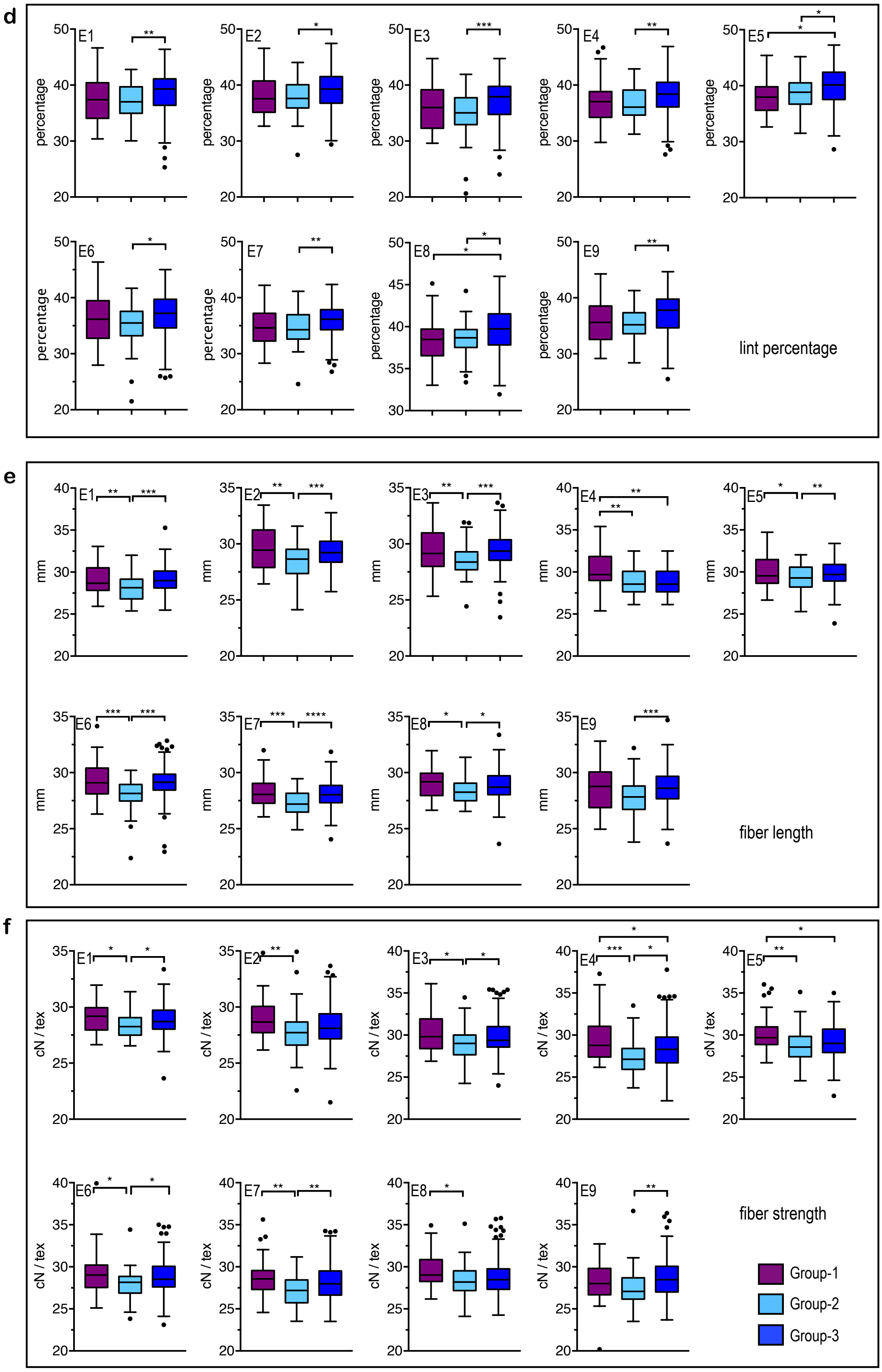
d
E1
E2
E3
E4
E5
E6
E7
E8
E9
percentage
lint percentage
e
mm
fiber length
f
cN / tex
fiber strength
Group-1
Group-2
Group-3

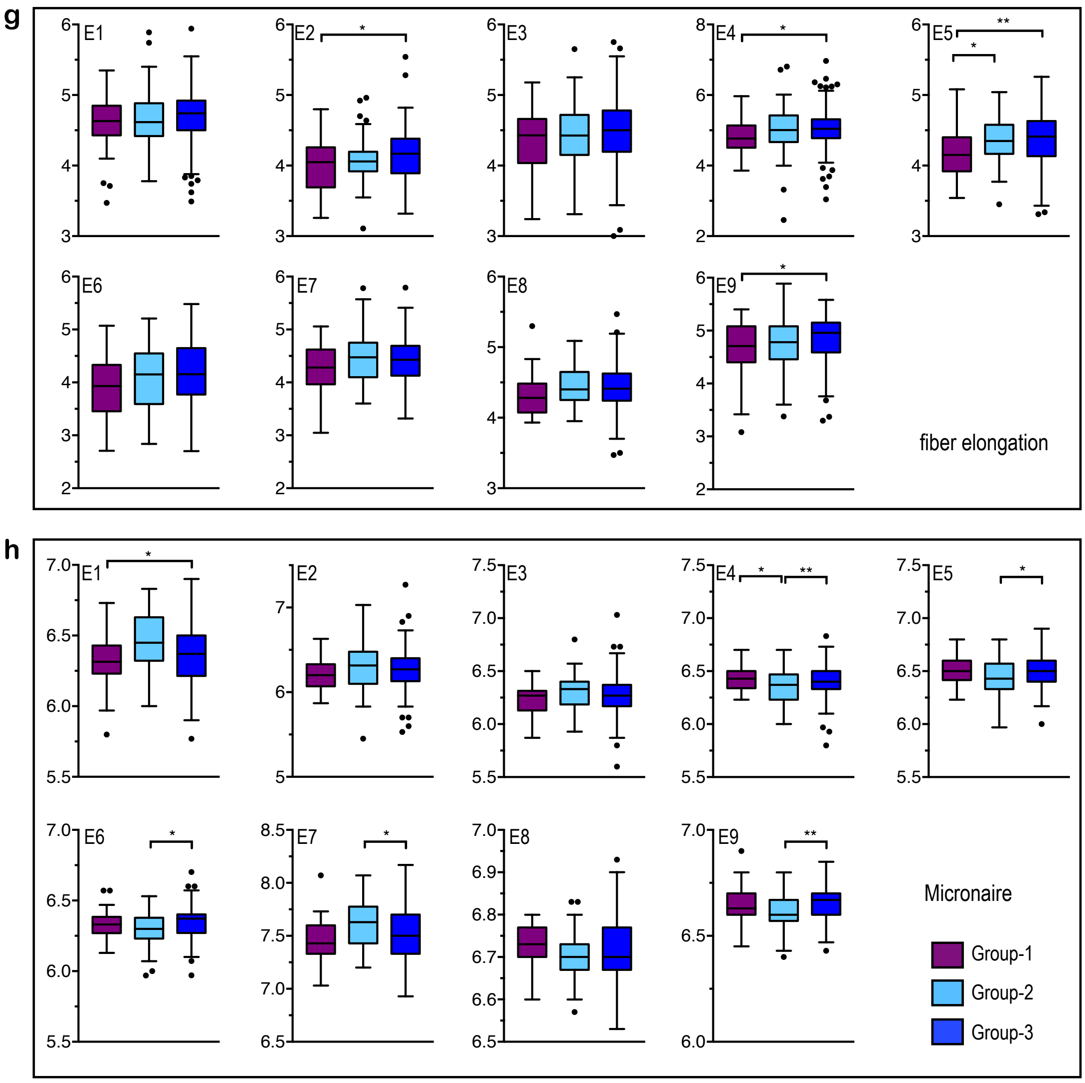

Supplementary Figure S2 Comparison of investigated traits in nine environments among groups. In the box plots, the center line, box limits, and whiskers indicates median, upper and lower quartiles, and 1.5× interquartile range, respectively. Points show outliers. One to three asterisks indicated significant level was at 5%, 1% and 0.1% in Dunn's multiple comparison test, respectively. (a) development stage, (b) boll weight, (c) seed index, (d) lint percentage, (e) fiber length, (f) fiber strength, (g) fiber elongation, (h) micronaire. E1, Anyang, 2007, E2, Nanjing, 2007, E3, Akesu, 2007, E4, Anyang, 2008, E5, Nanjing, 2008, E6, Akesu, 2008, E7, Anyang, 2009, E8, Nanjing, 2009, E9, Akesu, 2009.

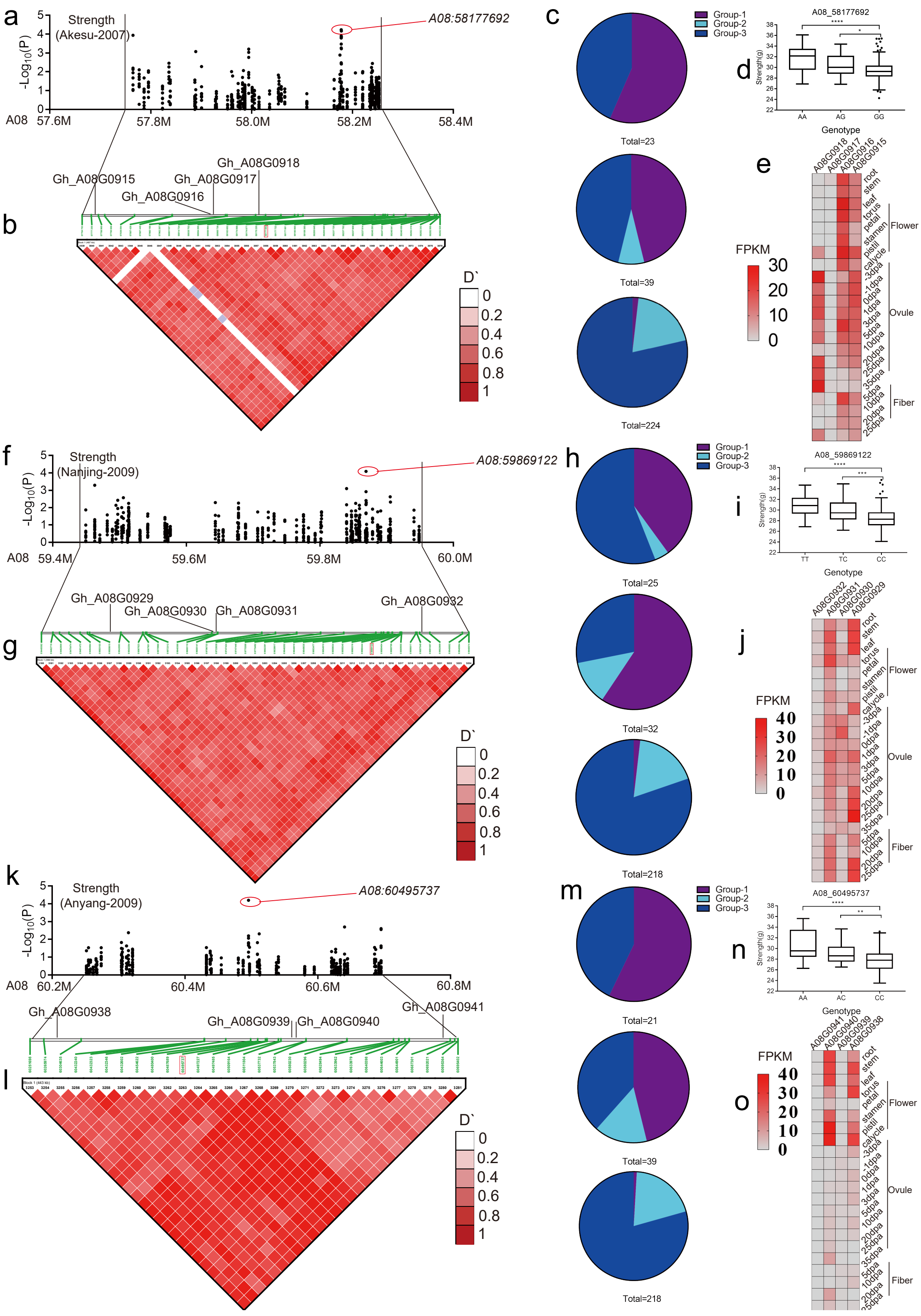

Supplementary Figure S3 The trait-associated loci within population divergence regions on A08. The local Manhattan plots (a, f, k) of GWAS for fiber strength, the strongest signals were marked by red circles. (b, g, i) LD blocks and genes in these regions, the positions of

strongest signals were labeled by red rectangles. The pie charts indicated the grouping for different genotypes of strongest signals (c, h, m). Box plots for development stage plotted as strongest signals (d, i, n), 1-4 asterisks indicated significant level was at 5%, 1%, 0.1% and 0.01% in Dunn's multiple comparison test, respectively. The heatmaps indicated gene expression profile in each block (e, j, o), respectively. FPKM, Fragments Per Kilobase per Million.

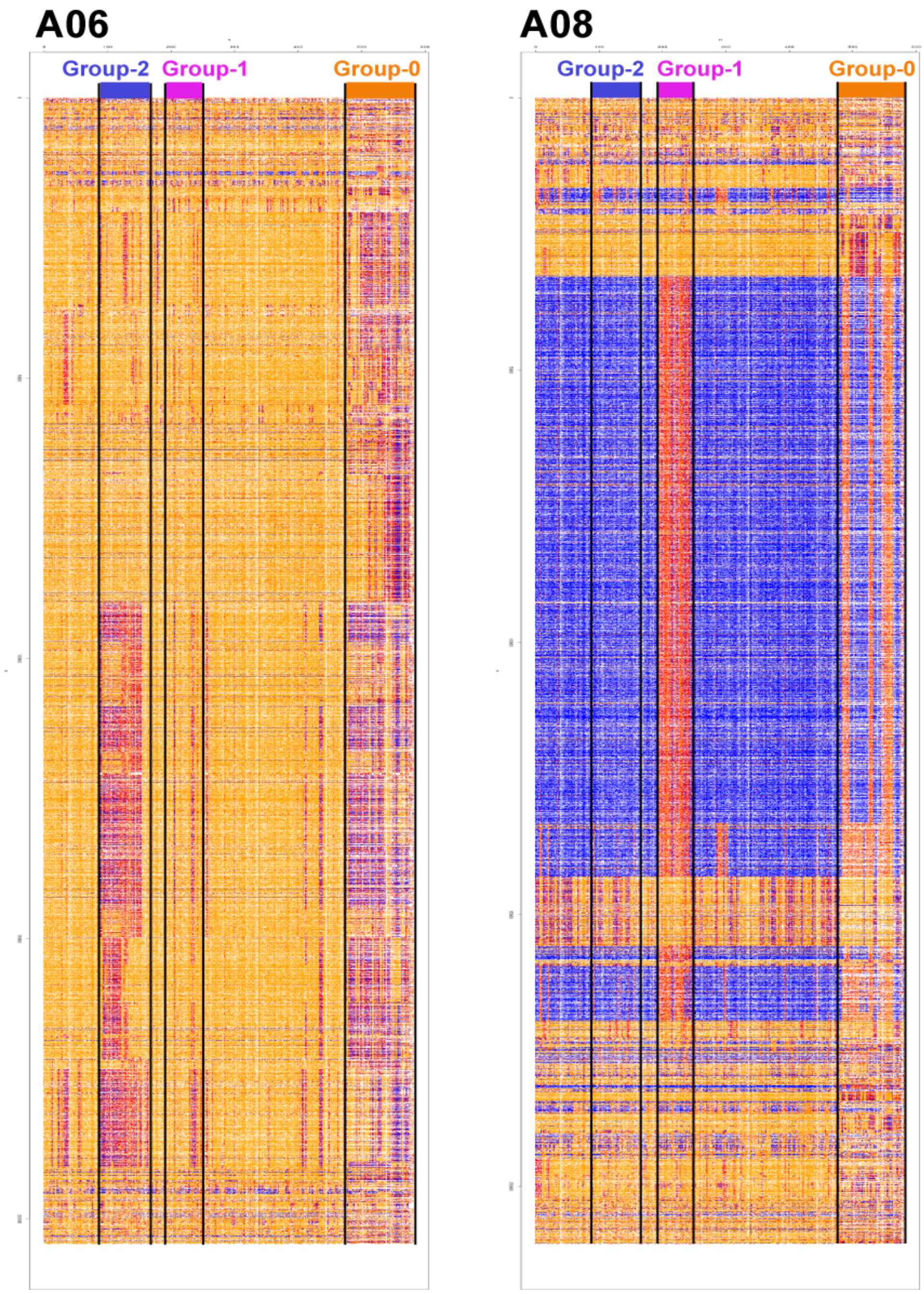

Supplementary Figure S4 Genotypes of the differential regions between populations identified on chromosome A06 and A08.

The x-axis represents all samples (the order is consistent with Fig 1C), and the y-axis represents the SNPs (consistent with their position on chromosomes from top to bottom). The genotypes included genotypes identical to the reference genome (orange), genotypes showing changes (blue), genotypes showing heterozygosis (red) and missing genotypes (white).

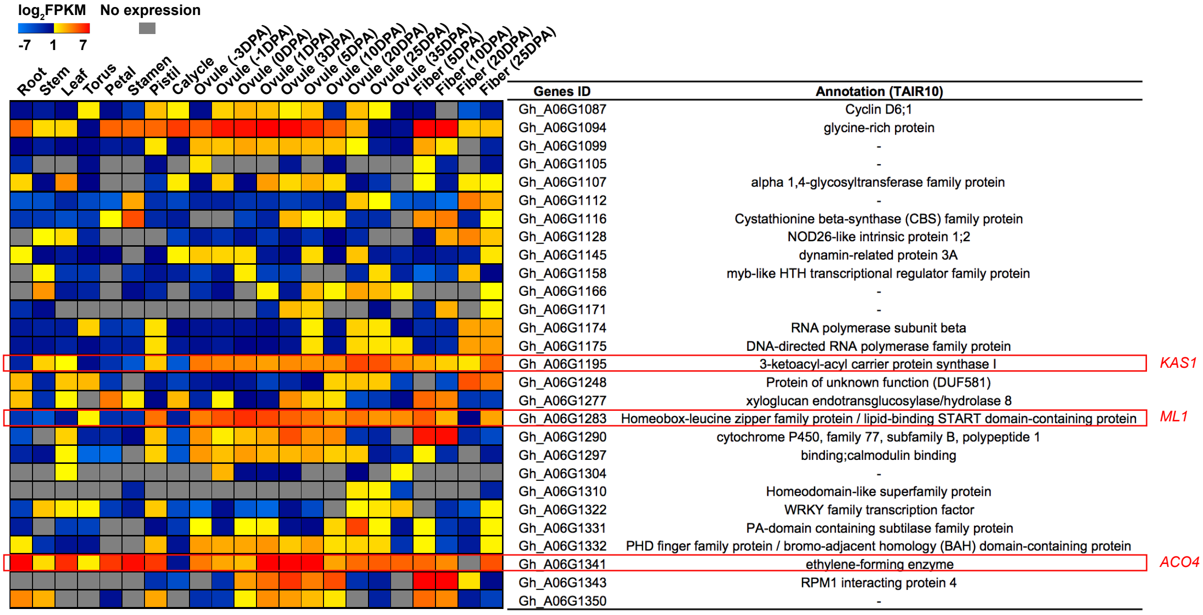

Supplementary Figure S5 Specifically expressed genes in the divergent genomic region of A06.

A total of 28 genes with ovule- or fiber-specific expression were selected from the divergent genomic region of A06. The gene expression profiles of various samples are shown in the left panel, and the gene IDs and annotations are shown in the right panel. Three functionally characterized genes are indicated by red boxes: *KAS1*, *3-ketoacyl-acyl carrier protein synthase I* (*AT5G46290*); *ML1*, *meristem layer 1* (*AT4G21750*); and *ACO4*, *1-aminocyclopropane-1-carboxylate oxidase* (*AT1G05010*). FPKM, fragments per kilobase of transcript sequence per millions base pairs.

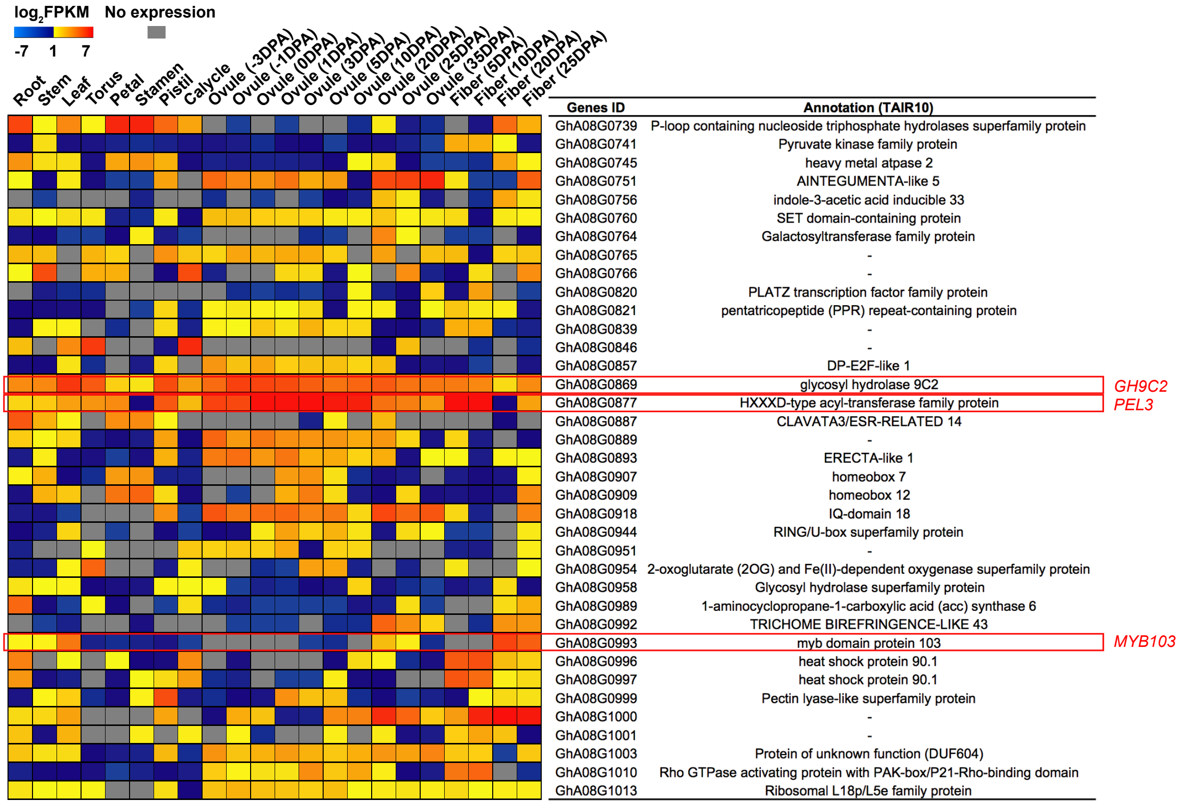

Supplementary Figure S6 The specifically expressed genes in the divergent genomic region on A08. Total 37 ovule or fiber specifically expressed genes were selected from the divergent genomic region on A08. The gene expression profiles in various samples are shown in left panel, and the gene IDs and annotations are shown in right panel. Three functionally characterized genes are marked by red boxes, *GH9C2*, *glycosyl hydrolase 9C2* (*AT1G64390*), *PEL3*, *permeable leaves3* (*AT5G23940*), *MYB103, myb domain protein 103* (*AT1G63910*). FPKM, fragments per kilobase of transcript sequence per millions base pairs.
